# Aldo-Keto Reductase Family 1 Member B10 (AKR1B10) overexpression in tumors predicts worse overall survival in hepatocellular carcinoma

**DOI:** 10.7150/jca.32768

**Published:** 2019-08-27

**Authors:** Jia Shi, Lixiang Chen, Yi Chen, Yunfei Lu, Xiaorong Chen, Zongguo Yang

**Affiliations:** 1 Department of Integrative Medicine, Shanghai Public Health Clinical Center, Fudan University, Shanghai 201508, China.; 2 Department of Laboratory Animal, Shanghai Public Health Clinical Center, Fudan University, Shanghai 201508, China.; 3 Department of Hepatobiliary Surgery, Shanghai Public Health Clinical Center, Fudan University, Shanghai 201508, China.

**Keywords:** AKR1B10, hepatocellular carcinoma, survival, outcome

## Abstract

Overexpression of AKR1B10 correlated with tumorigenesis of many human malignancies; however, the prognostic value of AKR1B10 expression in patients with hepatocellular carcinoma (HCC) still remains controversial. In this analysis, AKR1B10 expression in HCC tumors were evaluated in GEO, TCGA and Oncomine databases, and a survival analysis of AKR1B10 based on TCGA profile was performed. We found that AKR1B10 was significantly overexpressed in tumors compared with nontumors in 7 GEO series (GSE14520, GSE25097, GSE33006, GSE45436, GSE55092, GSE60502, GSE77314) and TCGA profile (all *P* < 0.05). Meta-analysis in Oncomine database revealed that AKR1B10 was significantly upregulated in cirrhosis, liver cell dysplasia and HCC compared with normal tissues (all *P* < 0.05). Kaplan-Meier analysis demonstrated that high AKR1B10 in tumors were significantly associated with worse overall survival (OS) in HCC patients (*P* < 0.05). Subgroup analysis showed that AKR1B10 overexpression were associated with poor 1-year, 3-year and 5-year OS (all *P* < 0.05). In addition, prognostic values of AKR1B10 upregulation for OS were more significant in HCC with hepatitis-virus-free (*P* = 0.00055), White race (*P* = 0.0029) and alcohol-free (*P* = 0.013), and both in male and female (*P* = 0.014 and *P* = 0.034, respectively). In conclusion: AKR1B10 was upregulated in tumors and correlated with worse OS in HCC patients.

## Introduction

Liver cancer, comprising 75%~85% cases of hepatocellular carcinoma (HCC), is predicted to be the sixth most commonly diagnosed cancer and the fourth leading cancer related deaths worldwide in 2018 1-4. In addition, the incidence of HCC will continue to rise over the next 10-20 years and to peak around 2030 based on a SEER registry projects study 3, 5. There has been a marked increase in HCC related annual death rates during the past two decades 3. Precise estimation of prognosis plays a critical role in treatment decision in HCC patients. Thus, to identify reliable prognostic biomarkers and to reveal HCC target for treatment is urgently required 6, 7.

Belongs to the aldo-keto reductase superfamily 8, 9, AKR1B10 is predominantly expressed in the small intestine, colon, liver, thymus and adrenal gland 8, 10. Upregulation of AKR1B10 has been observed in various human malignancies, including non-small cell lung carcinoma 11, pancreatic cancer 12, breast cancer 13, 14, oral squamous cell carcinoma 15, nasopharyngeal carcinoma 16 and HCC 17, 18. Conversely, underexpression of AKR1B10 has been observed in colon 19, gastric 20, endometrial cancer 21 and head and neck cancers 22. However, AKR1B10 in HCC has been controversially discussed. Some researches revealed that negatively correlated with serum alpha-fetoprotein (AFP) level, high AKR1B10 expression was found to be a favorable factor of overall survival and disease-free survival in patients with hepatitis B virus-related HCC 23-25. Another study found that high AKR1B10 expression was an independent risk factor for HCC development 26 and significantly correlated with liver damage^25^. Thus, to reevaluate the prognostic value of AKR1B10 in HCC patients is of great importance.

To help elucidate the possible relationship between AKR1B10 expression and HCC outcomes, we identified the AKR1B10 expression in GEO, Oncomine and TCGA databases and performed a survival analysis based on TCGA profile, in the hope of providing useful insights into hepatocarcinogenesis and aggressiveness.

## Materials and Methods

### Data resource and Description

Seven expression microarray series GSE14520, GSE25097, GSE33006, GSE45436, GSE55092, GSE60502 and GSE77314 containing HCC tumor and nontumor samples were downloaded from the Gene Expression Omnibus dataset (GEO, https://www.ncbi.nlm.nih.gov/geo/). Platforms and samples of GEO series were summarized in Table 1.

TCGA liver hepatocellular carcinoma mRNA normalized counts data derived from RNAseq Htseq platform were downloaded from Genomic Data Commons Data Portal (https://cancergenome.nih.gov/). TCGA RNAseq data contains 424 samples with 374 primary tumor and 50 solid tissue normal samples. RTCGAToolbox 27 and edgeR 28, 29 packages were used to identify AKR1B10 expression between tumor and normal samples.

### Bioinformatics analysis for identifying AKR1B10 expression

Raw.CEL files of the microarray from each GEO dataset were normalized by quantile method of Robust Multichip Analysis (RMA) from R affy package 30. Gene expression of AKR1B10 was performed by tumor and normal comparison from R Limma package 31. Gene expression data of GSE77314 in XLSX form was downloaded from GEO dataset directly.

Studies compared AKR1B10 between cancer and normal samples in liver cancer were selected with threshold by p-Value ≤ 1E-4, fold change ≥ 2 and top 10% gene rank in Oncomine database (https://www.oncomine.org/).

### Survival analysis

Liver Hepatocellular Carcinoma (TCGA, Provisional) database in cBioPortal for cancer genomics web service was used for identifying AKR1B10 for predicting the overall survival (OS) of HCC patients 32, 33. AKR1B10 Mrna expression levels calculated by log2 calculation were compared based on clinical attribute in HCC patients. To evaluate associations between candidate biomarkers and survival and linic-pathological features in HCC patients, gene data with Z scores and clinical data of HCC patients in Liver Hepatocellular Carcinoma (TCGA, Provisional) database were downloaded from cBioPortal and matched using VLOOKUP index in EXCEL. After excluding ten patients with liver histology of hepatocholangiocarcinoma (n = 7) and fibrolamellar carcinoma (n = 3) and six patients without gene expression levels, 361 HCC patients were included in the analysis. Additionally, the Kaplan Meier plotter online service (http://kmplot.com/analysis/) 34 was used for subgroup survival analysis of AKR1B10 with median cutoff in HCC patients.

### Protein-protein interaction and pathway/biological process enrichment

Protein-Protein Interaction analysis (PPI) for AKR1B10 were performed by STRING database (https://string-db.org/). KEGG pathway and GO biological process enrichment analysis were conducted using Gene Set Enrichment Analysis (GSEA, http://software.broadinstitute.org/gsea/index.jsp). To investigate gene sets, AKR1B10 interactive genes were uploaded to Molecular Signatures Database in GSEA. A false discovery rate *P* value cut‑off of <0.05 was set as the screening condition.

### Statistical analysis

Differences of gene expression between the individual groups were analyzed using student *t* test, Mann-Whitney *U*-test, Chi-square test and Ridit analysis based on variables types. PASW Statistics software version 23.0 from SPSS Inc. (Chicago, IL, USA) was used. A two-tailed *P* < 0.05 were considered significant for all tests.

## Results

### AKR1B10 expression comparison

**Figure 1:**
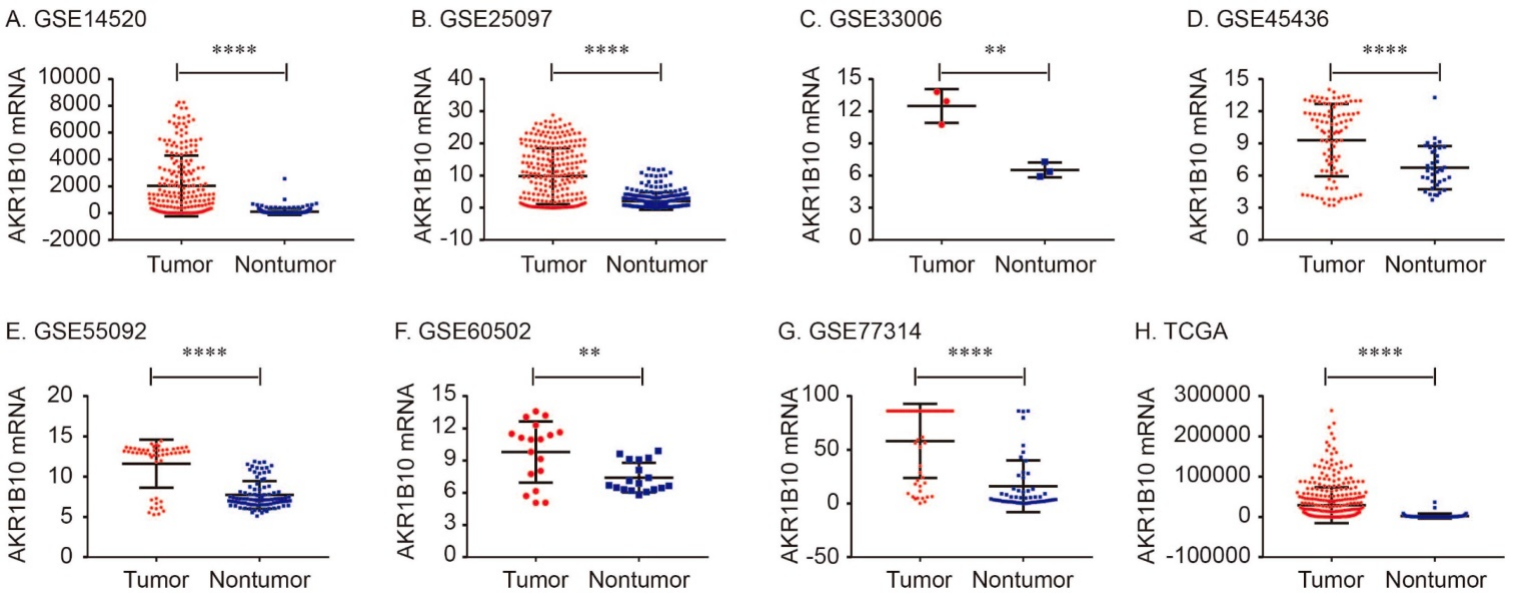
AKR1B10 mRNA expression levels between tumor and nontumor tissues in HCC patients in GEO database series including GSE14520 (A), GSE25097 (B), GSE33006 (C), GSE45436 (D), GSE55092 (E), GSE60502 (F), GSE77314 (G) and TCGA database (H).

The details of GEO series included in this analysis were summarized in Table 1. As shown in Figure 1, AKR1B10 Mrna was significantly overexpressed in GSE14520, GSE25097, GSE33006, GSE45436, GSE55092, GSE60502. GSE77314 and TCGA datasets (all *P* < 0.01, Figure 1).

**Figure 2:**
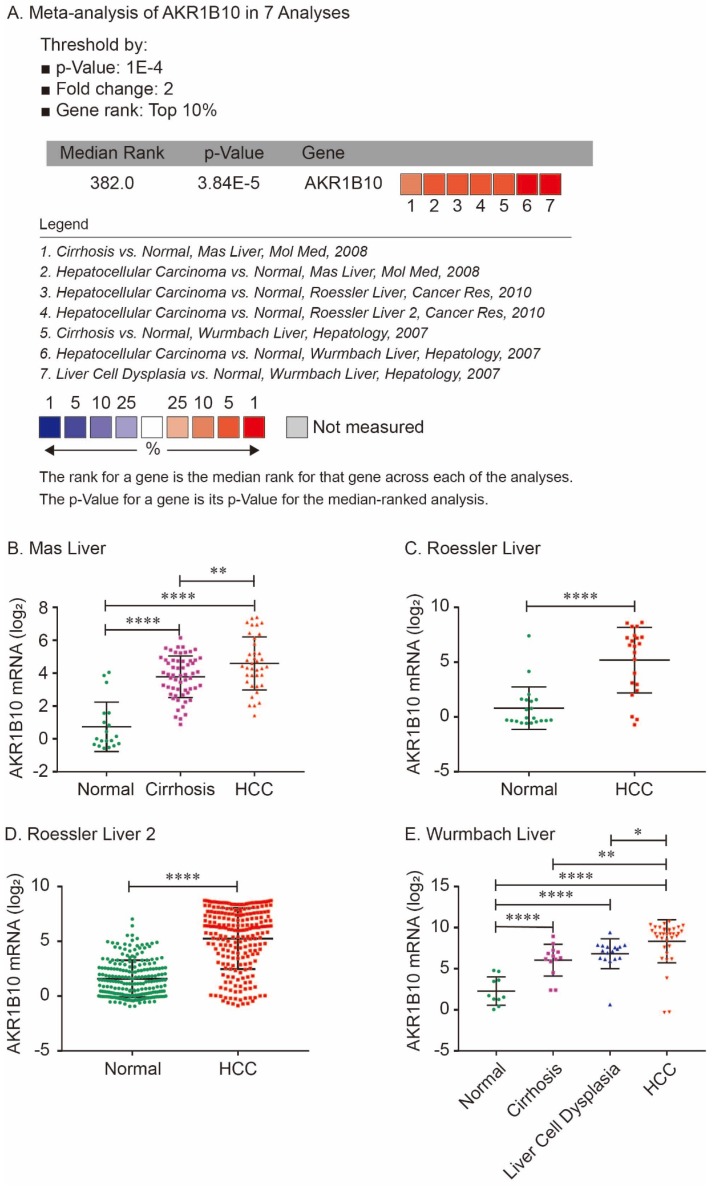
Comparison of AKR1B10 mRNA expression levels across 7 analyses in Oncomine database. Meta-analysis of AKR1B10 expression in 7 analyses (A); AKR1B10 levels in normal, cirrhosis and HCC tissues in Mas Liver (B); AKR1B10 levels in normal and HCC tissues in Roessler Liver (C) and Roessler Liver 2 (D); AKR1B10 levels in normal, cirrhosis, liver cell dysplasia and HCC tissues in Wurmbach Liver (E).

For validation, we performed meta-analysis of AKR1B10 expression in 7 analyses with threshold by p-Value ≤ 1E-4, fold change ≥ 2 and top 10% gene rank in Oncomine database. As shown in Figure 2, compared with that in normal liver tissues, AKR1B10 was significantly upregulated in cirrhosis (*P* < 0.0001, Figure 2A, 2B and 2E), liver cell dysplasia (*P* < 0.0001, Figure 2A and 2E) and HCC tumors (*P* < 0.0001, Figure 2A - 2E). Additionally, AKR1B10 was significantly higher in HCC tumor tissues than that in cirrhosis (*P* < 0.01, Figure 2B and 2E) and in liver cell dysplasia (*P* < 0.05, Figure 2E).

### Associations between AKR1B10 and OS in HCC patients

**Figure 3:**
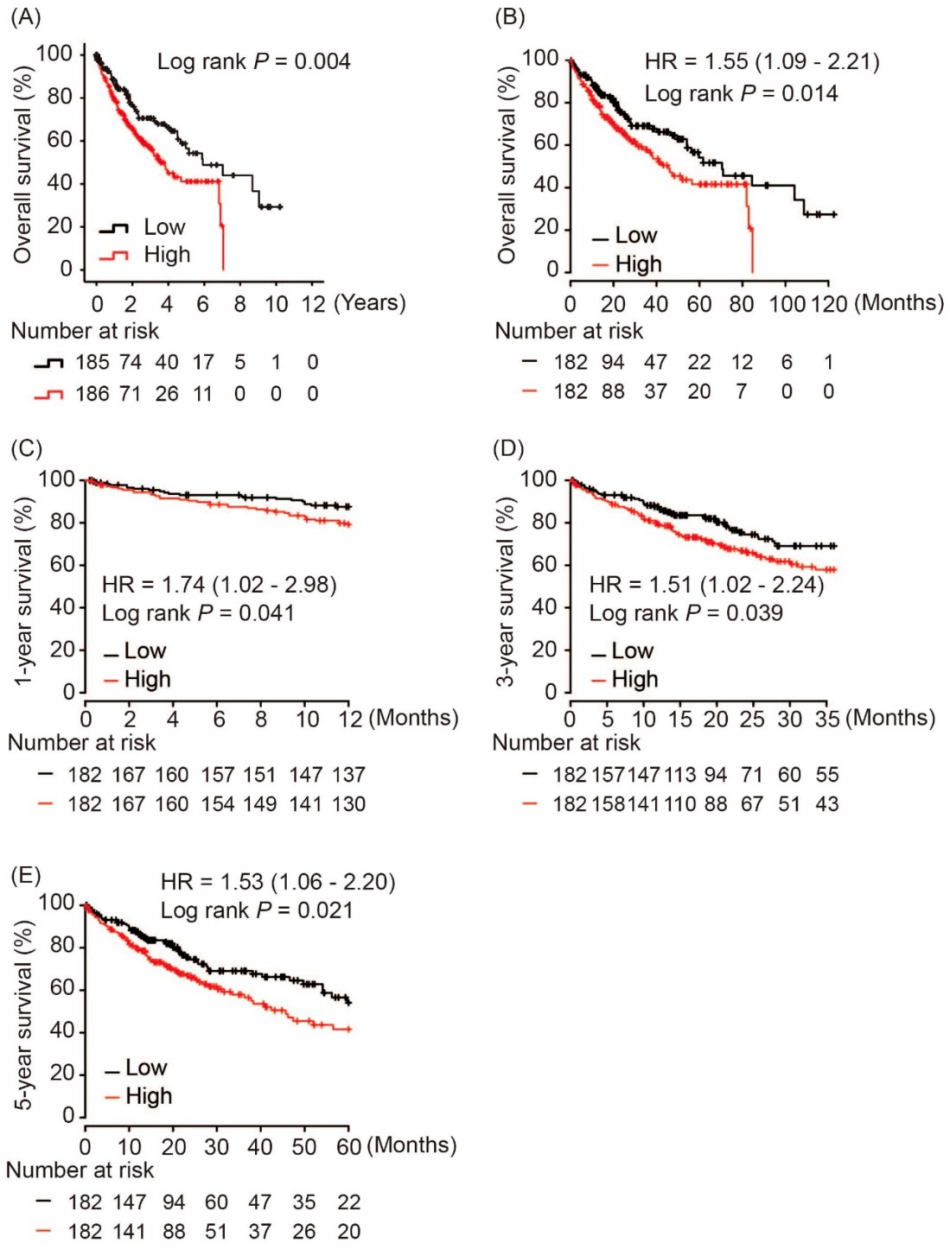
Overall survival of HCC patients grouped by AKR1B10 median cutoff in TCGA database (A, B); 1-year (C), 3-year (D) and 5-year overall survival comparison between AKR1B10 high and low groups.

Using hash and survival packages in R program, we performed Kaplan-Meier analysis of AKR1B10 and OS in HCC patients, which showed that AKR1B10 overexpression in tumor tissues was significantly associated with poor OS in HCC patients (log rank *P* = 0.004, Figure 3A). A consistent result was validated in Kapan-Meier Plotter online service as shown in Figure 3B (log rank* P* = 0.014, Figure 3B). Moreover, subgroup analysis revealed that AKR1B10 upregulation in tumors was risk factor for 1-year, 3-year and 5-year OS in HCC patients (HR = 1.74, log rank *P* = 0.041; HR = 1.51, log rank *P* = 0.039 and HR = 1.53, log rank *P* = 0.021, respectively, Figure 3C - 3E).

**Figure 4:**
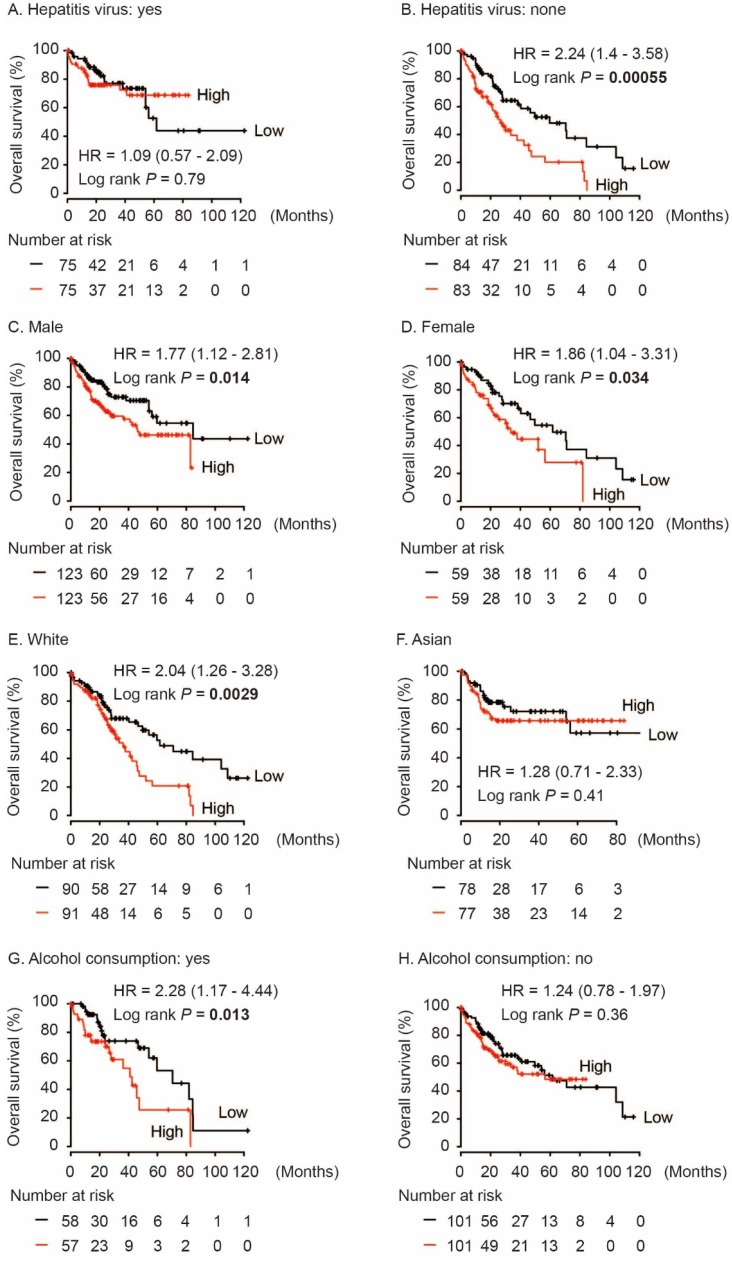
Subgroup analyses of overall survival comparison in different population [hepatitis virus infection status (A, B), gender (C, D), race (E, F) and alcohol consumption (G, H)] with AKR1B10 median cutoffs in HCC patients.

In addition, we performed subgroup survival analysis in different population. As shown in Figure 4, high AKR1B10 level significantly contributed to worse OS in HCC patients without hepatitis virus infection (HR = 2.24, log rank *P* = 0.00055, Figure 4B). And, AKR1B10 overexpression was significantly associated with OS both in male and female (HR = 1.77, log rank *P* = 0.014 and HR = 1.86, log rank *P* = 0.034, respectively, Figure 4C and 4D). AKR1B10 was risk factor for OS in White HCC (HR = 2.04, log rank *P* = 0.0029, Figure 4E) and in cases with alcohol consumption (HR = 2.28, log rank *P* = 0.013, Figure 4G).

**Figure 5:**
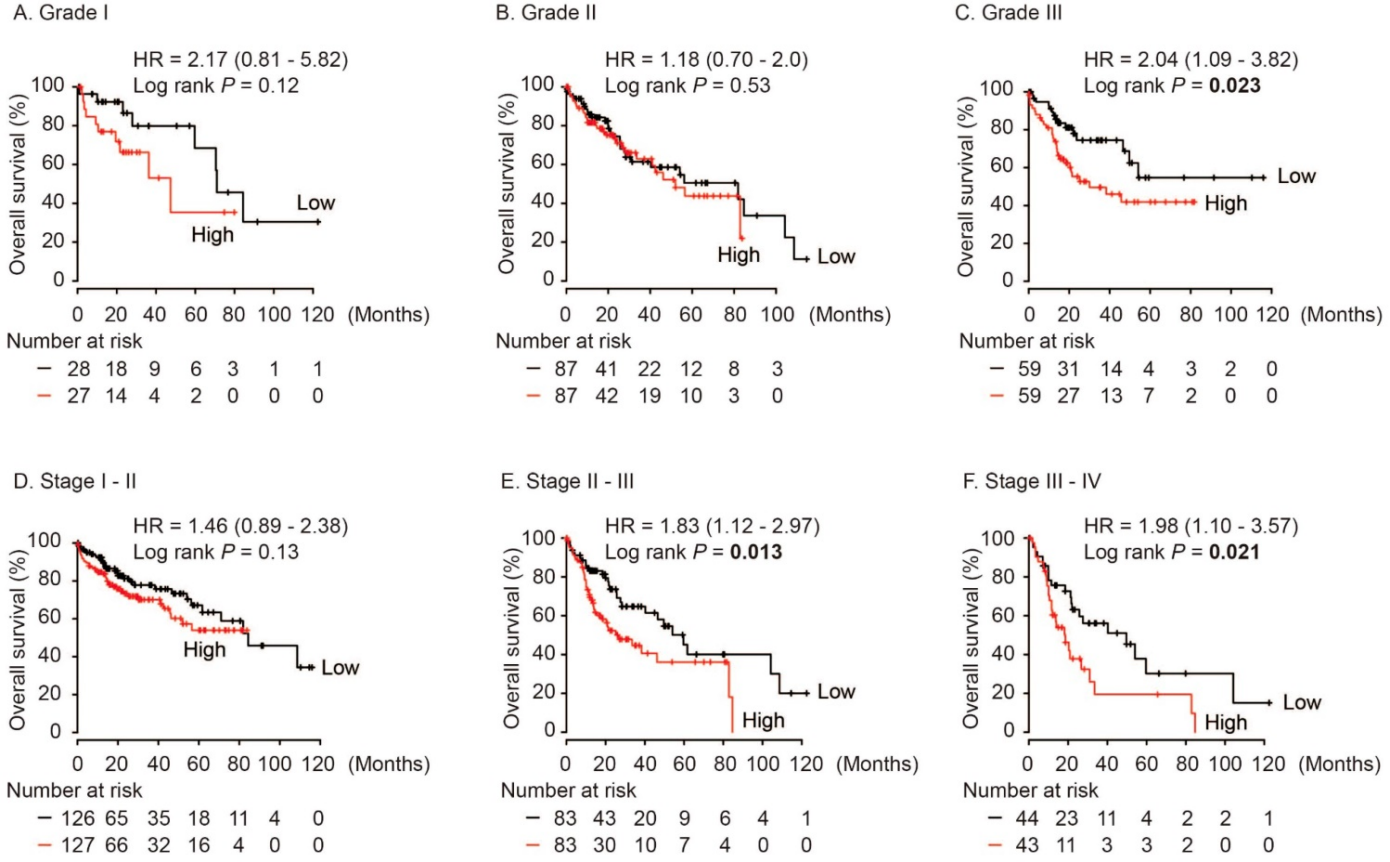
Overall survival of HCC patients grouped by AKR1B10 median in different grades (A, B, C) and stages (D, E, F).

In HCC patients with neoplasm histologic Grade III, AKR1B10 was significantly associated with poor OS (HR = 2.04, log rank *P* = 0.023, Figure 5C), while no differences were found in HCC cases with Grade I and Grade II (log rank *P* > 0.05, Figure 5A and 5B). Moreover, AKR1B10 overexpression was significantly contributed to poor OS in HCC patients with stage II-III (HR = 1.83, log rank *P* = 0.013, Figure 5E) and stage III-IV (HR = 1.98, log rank *P* = 0.021, Figure 5F).

### Association between AKR1B10 and clinico-pathological features in HCC patients

As shown in Table 2, more male cases in AKR1B10 high group (76.2% vs. 58.9%, *P* < 0.001) and patients in AKR1B10 high group were significantly older than those in AKR1B10 low group (62.0yr vs. 59.5yr, *P* = 0.031). More HCC cases had family history of cancer in AKR1B10 high group than those in AKR1B10 low group (43.1% vs. 30.0%, *P* = 0.01). More HCC cases had hepatocarcinoma risk factors (especially alcohol consumption) in AKR1B10 high group than those in AKR1B10 low group (*P* = 0.006). As we expected, HCC patients in AKR1B10 high group suffered from significantly advanced Ishak fibrosis status and advanced hepatic inflammation (*P* = 0.036 and *P* = 0.033, respectively).

#### PPI and KEGG/GO biological process enrichment

**Figure 6:**
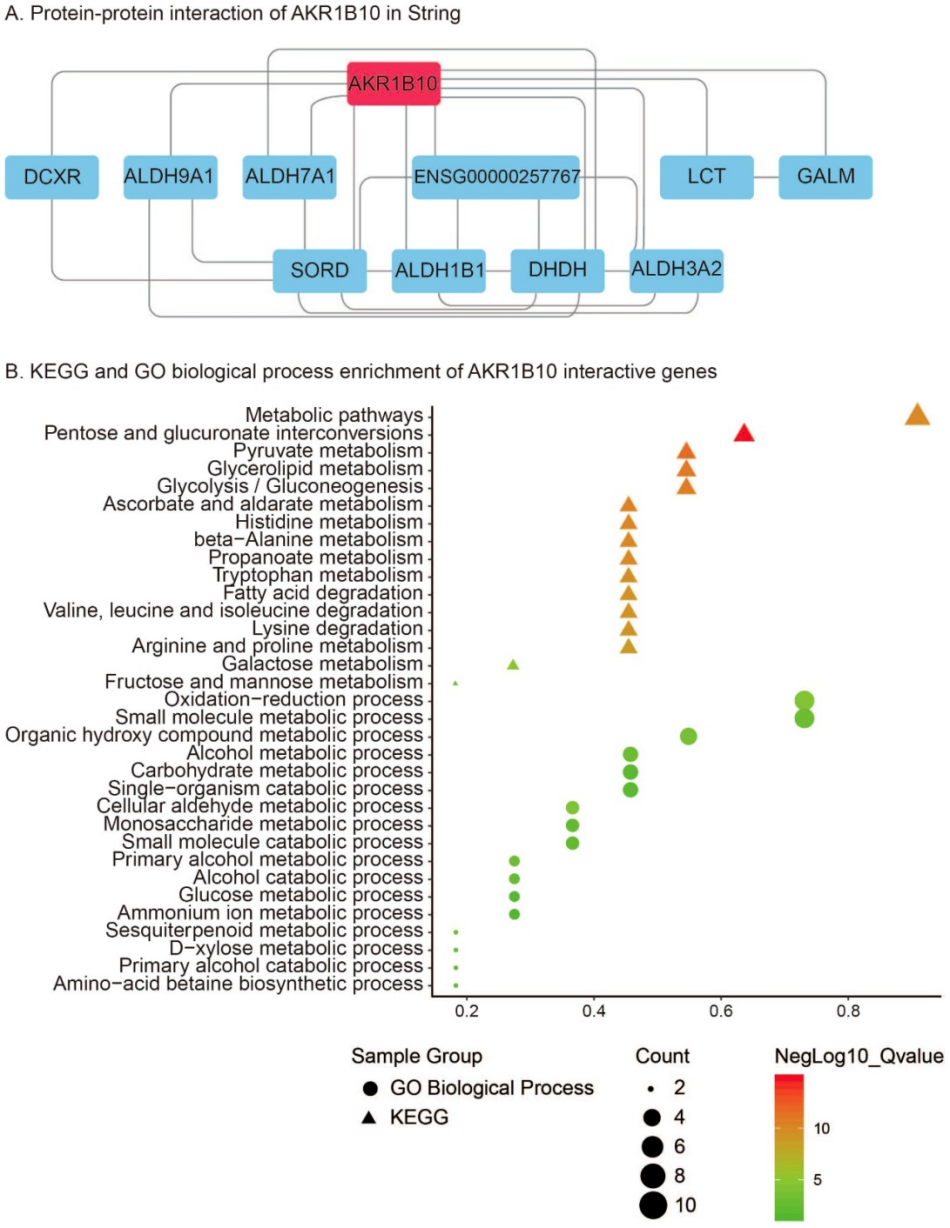
Protein-protein interaction of AKR1B10 using String analysis (A); KEGG and GO biological process enrichment of its interactive genes (B)

PPI analysis using STRING revealed that 10 genes including DCXR, ALDH9A1, ALDH7A1, ENSG00000257767, LCT, GALM, SORD, ALDH1B1, DHDH and ALDH3A2 were interacted with AKR1B10 (Figure 6A). KEGG pathway enrichment of AKR1B10 interactive genes showed that metabolic pathways, pentose and glucuronate interconversions, pyruvate metabolism, glycerolipid metabolism, glycolysis / gluconeogenesis, ascorbate and aldarate metabolism, histidine metabolism, beta-Alanine metabolism, propanoate metabolism, tryptophan metabolism, arginine and proline metabolism, fatty acid degradation, etc were the most enriched pathways (Figure 6B). Additionally, oxidation-reduction process, metabolic process including small molecular, organic hydroxy compound, alcohol, carbohydrate, single-organism, cellular aldehyde, glucose, ammonium ion, etc were most enriched GO biological process of AKR1B10 interactive genes (Figure 6B).

## Discussion

Over the past few years, new data have recently emerged that implicate AKR1B10 in tumor carcinogenesis in different systemic malignancies 35. AKR1B10 is expressed at lower levels in normal liver^8^, ^10^ and overexpressed in HCC tumors 22, 25. Matkowskyj K et al reported that AKR1B10 was overexpressed in 97% of HCC, with minimal to no expression in adjacent hepatic tissue, while hepatic adenomas and focal nodular hyperplasia did not exhibit expression of AKR1B10 17. A small sample study demonstrated that increased expression of AKR1B10 in moderately-differentiated HCC compared with well-differentiated HCC, poorly-differentiated HCC, and liver cirrhosis 18. Consistent with previous reports, we found that AKR1B10 was significantly upregulated in cirrhosis, liver cell dysplasia and HCC tumors compared with normal livers. Additionally, AKR1B10 was significantly higher in HCC tumor tissues than that in cirrhosis and in liver cell dysplasia. Hence, we assumed that AKR1B10 is emerging as a biomarker to distinguish hepatocellular carcinoma from benign liver lesions.

However, roles of AKR1B10 in hepatocarcinogenesis are still controversial. In a retrospective study of 168 cases, Schmitz K et al found that loss of AKR1B10 expression correlates with increased proliferative activity. A poorer prognosis in AKR1B10-negative HCCs was revealed compared with patients with strongly positive HCCs 36. In 110 patients with hepatitis B virus-related HCC, high AKR1B10 expression was negatively correlated with serum AFP level. Patients with high AKR1B10 expression had significantly higher DFS than those with low expression within 2 years after liver resection. High AKR1B10 expression was found to be a favorable factor of early recurrence and OS in HCC patients 23. In 255 HCC patients who underwent curative hepatectomy, high AKR1B10 expression was significantly associated with a lack of invasion of the major portal vein, a lack of intrahepatic metastasis, lower tumor stages, and lower AFP levels, which was found to be an independent predictor of both longer recurrence-free survival and longer disease-specific survival 24.

Conversely, our results demonstrated that high AKR1B10 in tumors were significantly associated with worse OS in HCC patients. AKR1B10 overexpression were associated with poor 1-year, 3-year and 5-year OS. A series publication enhanced our findings. Murata A et al reported that high AKR1B10 expression was an independent risk factor for HCC development in chronic hepatitis C patients. During the follow-up period after viral eradication, patients expressing high levels of AKR1B10 expressed markedly higher levels of alanine aminotransferase and AFP than did patients exhibiting low AKR1B10 expression 26. The oncogenetic functions of AKR1B10 in HCC tumor growth were validated in HCC cell lines SMMC-7721, HepG2 and Hep3B. Knockdown of AKR1B10 through shRNA in Hep3B cells showed significantly induced cell cycle arrest and inhibited cell growth 37. ShRNA-mediated silencing of AKR1B10 expression in HCC cells resulted in increased cell apoptosis, decreased colony formation and size, and enhanced cytoreductive response following exposure to doxorubicin chemotherapy 17. A recent study identified that AKR1B10 is a novel downstream target of interleukin-1 receptor-associated kinase 1 (IRAK1), which was found to be overexpressed in HCC and significantly correlated with IRAK1 expression. Knockdown of AKR1B10 negated IRAK1-induced tumor-initiating cells functions 38.

Functionally, AKR1B10 exerts a protective role through eliminating oxidative and carbonyl stresses and promoting epithelial proliferation for damage repair in inflammation. The KEGG pathway and GO biological process of AKR1B10 interactive genes in our study revealed that AKR1B10 should develop metabolic pathways and oxidation-reduction process. Unfortunately, we could not perform experimental research for probing potential oncogenic mechanisms of AKR1B10 in HCC development. And, no our own follow-up data of HCC patients were available. Considered the current controversial publications, we suggest a meta-analysis to evaluate the links between AKR1B10 and HCC prognosis. Even though, considered previous reports, we cautiously drew the hypothesis that AKR1B10 overexpression contributed to unfavorable prognosis in HCC patients.

## Figures and Tables

**Table 1:** Details of GEO series included in this analysis

GEO series	Contributor(s)	Tumor	Nontumor	Platform
GSE14520	Roessler S et al, 2009	222	212	Affymetrix Human Genome U133A 2.0 Array / Affymetrix HT Human Genome U133A Array
GSE25097	Zhang C, 2010	268	243	Rosetta/Merck Human RSTA Affymetrix 1.0 microarray, Custom CDF
GSE33006	Huang Y et al, 2011	3	3	Affymetrix Human Genome U133 Plus 2.0 Array
GSE45436	Hsieh J, 2013	97	37	Affymetrix Human Genome U133 Plus 2.0 Array
GSE55092	Melis M et al, 2014	49	91	Affymetrix Human Genome U133 Plus 2.0 Array
GSE60502	Kao KJ, 2014	18	18	Affymetrix Human Genome U133A Array
GSE77314	Hou G et al, 2016	50	50	Illumina Genome Analyzer (Homo sapiens)

**Table 2:** Characteristics of HCC patients between AKR1B10 high and low groups

Variables	AKR1B10 expression level		*P* value
	Low (n = 180)	High (n = 181)	
Gender, male (%)	106 (58.9)	138 (76.2)	< 0.001
Age, median (IQR), years	59.5 (20)	62 (15)	0.031
BMI, kg/m^2^, n (%)			0.132
<18.5	14 (7.8)	7 (3.9)	
18.5~24.99	79 (43.9)	73 (40.3)	
25~29.99	36 (20.0)	52 (28.9)	
>30	35 (19.4)	32 (17.7)	
NA	16 (8.9)	17 (9.4)	
Race, n (%)			0.36
Asian	77 (42.8)	79 (43.6)	
White	91 (50.6)	85 (47.0)	
Black or African American	7 (3.9)	10 (5.5)	
American Indian or Alaska Native	2 (1.1)	0 (0)	
NA	3 (1.6)	7 (3.9)	
Tumor status, n (%)			0.923
With tumor	53 (29.4)	56 (30.9)	
Tumor free	115 (63.9)	112 (61.9)	
NA	12 (6.7)	13 (7.2)	
Family history of cancer, n (%)	54 (30.0)	78 (43.1)	0.01
Hepatocarcinoma risk factors*, n (%)			0.006
Hepatitis virus infection	55 (30.6)	56 (30.9)	
Alcohol consumption	48 (26.7)	67 (37.0)	
Non-alcoholic fatty liver disease	6 (3.3)	6 (3.3)	
No risk factors	54 (30.0)	32 (17.7)	
Other	8 (4.4)	10 (5.5)	
NA	9 (5.0)	10 (5.5)	
Neoplasm histologic grade, n (%)			0.054
G1	35 (19.4)	18 (9.9)	
G2	76 (42.2)	95 (52.5)	
G3	60 (33.3)	61 (33.7)	
G4	6 (3.3)	5 (2.8)	
NA	3 (1.7)	2 (1.1)	
AJCC stage, n (%)			0.203
I	84 (46.7)	83 (45.9)	
II	35 (19.4)	47 (26.0)	
III	48 (26.7)	33 (18.2)	
IV	4 (2.2)	3 (1.7)	
NA	9 (5.0)	15 (8.3)	
Vascular invasion, n (%)			0.999
Macro	8 (4.4)	8 (4.4)	
Micro	44 (24.4)	45 (24.9)	
None	99 (55.0)	101 (55.8)	
NA	29 (16.1)	27 (14.9)	
Child-pugh classification, n (%)			0.525
A	101 (56.1)	111 (61.3)	
B	9 (5.0)	12 (6.6)	
C	1 (0.6)	0 (0)	
NA	69 (38.3)	58 (32.0)	
AFP > 400ng/ml, n (%)	39 (21.7)	25 (13.8)	0.051
Platelet, median (IQR), ×10^3^/mm^3^	204.5 (182)	178 (136)	0.066
New tumor event after initial treatment, n (%)	46 (25.6)	47 (26.0)	0.929
Ishak fibrosis status, n (%)			0.036
No fibrosis	44 (24.4)	28 (15.5)	
Portal fibrosis	10 (5.6)	21 (11.6)	
Fibrous speta	11 (6.1)	17 (9.4)	
Nodular Formation/Incomplete Cirrhosis	3 (1.7)	6 (3.3)	
Cirrhosis	30 (16.7)	40 (22.1)	
NA	82 (45.6)	69 (38.1)	
Hepatic inflammation, n (%)			0.033
None	69 (38.3)	45 (24.9)	
Mild	43 (23.9)	54 (29.8)	
Severe	7 (3.9)	11 (6.1)	
NA	61 (33.9)	71 (39.2)	
Follow up, median (IQR), years	0.378 (1.8)	0.334 (1.3)	0.453

IQR, interquartile range; BMI, body mass index; AJCC, American Joint Committee on Cancer; AFP, alpha-fetoprotein; NA, not available. * Sum of all risk factors compared with no risk factors.
